# Peri-Implantitis Treatment on Microbial Decontamination

**DOI:** 10.3390/microorganisms13071681

**Published:** 2025-07-17

**Authors:** Maria Pia Di Palo

**Affiliations:** Department of Medicine, Surgery and Dentistry, University of Salerno, 84084 Salerno, Italy; mariapia140497@gmail.com

Peri-implantitis, as defined by the 2017 World Workshop on the Classification of Periodontal and Peri-Implant Diseases and Conditions, is a pathological condition affecting the tissues around dental implants, with inflammation in the outer peri-implant tissues and bone loss [1,2]. In turn, maintaining the health of the peri-implant tissues involves ensuring the absence of clinical signs of inflammation and radiographic evidence of additional bone loss after physiological remodeling [3,4,5,6].

Peri-implantitis microbial profile is characterized by a broader diversity with an increased abundance of bacteria of the Socransky et al. [7] red and orange complexes (such as *Porphyromonas gingivalis*, *Treponema denticola*, and *Tannerella forsythia*) compared to periodontitis [8]. The treatment of peri-implantitis aims to reduce the microbial load, decontaminate the dental implant surface, and eliminate peri-implant mucosal inflammation [9,10], thereby preserving the peri-implant bone, and can be divided into nonsurgical and surgical interventions [11,12,13].

Peri-implantitis nonsurgical treatment includes mechanical debridement, antiseptic adjuncts and antibiotics, and chemical and laser surface decontamination [14].

Local mechanical decontamination through manual instruments constitutes a prevalent approach, with curettes being the primary method, either utilized independently or in conjunction with ultrasonic scalers, air-powder devices, and titanium brushes [15]. Both surgical and non-surgical peri-implant mechanical decontamination showed limited efficacy in reducing tissue-invasive bacteria, such as *Fusobacterium nucleatum* [14].

Air polishing, particularly using amino acid glycine or sodium bicarbonate powders, was effective in completely removing bacterial plaque biofilms without causing significant damage to sandblasted and acid-etched titanium surfaces [16]. However, comprehensive disease resolution was not consistently achieved, and air polishing did not significantly improve bleeding on probing (BoP) or disease resolution compared to mechanical debridement at mucositis sites. Conversely, amino acid glycine and sodium bicarbonate powders, when repeatedly applied, were associated with the complete removal of bacterial plaque biofilms [17]. Furthermore, low-abrasive erythritol showed lower abrasiveness and smaller particle size compared to glycine air polishing and, due to its capability to bind antiseptic substances, has been suggested as suitable for submucosal biofilm removal. Recent data also indicate an inhibitory effect of erythritol on periodontopathogens, such as *Porphyromonas gingivalis*, highlighting its potential in supportive periodontal therapy [18].

Local chemical decontamination methods encompass chlorhexidine-based formulations, 3% hydrogen peroxide, citric acid, metronidazole gel, antimicrobial solutions (e.g., tetracycline hydrochloride and minocycline ointments), and enamel matrix derivative [15].

Contrary to expectations, supplementary chlorhexidine therapy alongside mechanical debridement did not improve probing depth (PD) outcomes in individuals with peri-implant mucositis or peri-implantitis compared to those undergoing mechanical debridement alone [19]. This lack of effect might stem from the variable substantivity of chlorhexidine between tooth and implant surfaces, coupled with the dependence of chlorhexidine adhesion on surface texture and drug concentration. Non-treated implant surfaces quickly release chlorhexidine, whereas prepared surfaces (sandblasting/acid etching) may exhibit better chlorhexidine uptake. Furthermore, concerning the use of chlorhexidine as an adjunct in peri-implantitis treatment, the evolving issue about the antimicrobial resistance to chlorhexidine should be considered [12].

The local administration of various tetracycline combinations (doxycycline and minocycline) alongside both surgical and non-surgical interventions demonstrated substantial improvements in BoP and PD. However, systemic antibiotics, including amoxicillin and metronidazole, either locally or systemically administered, showed a reduction of *Porphyromonas gingivalis* and *Tannerella forsythia* but did not confer significant therapeutic advantages, especially regarding PD, Gingival Index (GI), and clinical attachment level (CAL) [14,20,21,22].

Local probiotics administered for one month in peri-implant mucositis sites undergoing nonsurgical therapy exhibited a significant difference in BoP but not in PD. Although *Lactobacillus salivarius* probiotics were effective in counteracting primary pathogens associated with peri-implantitis, including *Porphyromonas gingivalis*, *Prevotella intermedia*, *Streptococcus aureus*, and *Streptococcus salivarius*, the available data do not substantiate the effective use of local probiotics in peri-implantitis management [23].

Local physical decontamination methods encompass lasers, photodynamic therapy, ozone therapy, and electrolytic current, as detailed in the systematic review with meta-analysis by Baima et al. (2022) [15].

Various lasers investigated exhibited distinct properties and settings, including wavelength, power, waveform, pulse duration, energy/pulse, energy density, exposure duration, angulation of energy toward targeted tissue, peak power of the pulse, and tissue properties [24,25]. Non-surgical approaches combined with erbium-doped yttrium aluminum garnet laser (Er: YAG), carbon dioxide (CO2), and diode laser applications demonstrated a higher short-term reduction in BoP, while surgical approaches yielded slight to no benefits in PD, Plaque Index (PI), BoP reduction, and CAL gain. However, a significant reduction in black-pigmented, Gram-negative anaerobic bacteria (such as *Aggregatibacter actinomycetemcomitans*, *Porphyromonas gingivalis*, and *Prevotella intermedia*) was observed [24].

Antimicrobial Photodynamic Therapy (aPDT), employing visible light to activate non-toxic photosensitizers, showed promise with various derivatives such as Phenothiazinium derivatives (Methylene Blue, Toluidine Blue), Porphyrin, chlorine, and phthalocyanine derivatives (TMPyP, XF-73, Chlorin-e6), Xanthene derivatives (Eosin Y, Erythrosine, Rose Bengal), Fullerene derivatives (Fullerene C60), Phanalenone derivatives (PNS, SAPYR), Riboflavin derivatives (Vitamin B2), and Curcumin derivatives (Curcumin). Despite advantages, such as the absence of antimicrobial resistance, minimal adverse effects, no systemic implications, and low cost, aPDT, when used adjunctively, did not exhibit evidence of improving outcomes compared to implant surface scaling/debridement alone. The effects of aPDT should be considered in future practice [26].

Ozone therapy, delivered as gas, water, oil, or gel, with gaseous formulation appearing most effective but also potentially hazardous if inhaled, swiftly transforms into oxygen molecules, thereby inactivating bacteria (particularly against *Porphyromonas gingivalis*) [6], fungi, and viruses, and controlling bleeding. Studies on the effectiveness of ozone, with or without mechanical debridement, yielded varying results [27].

Implantoplasty has demonstrated a significant enhancement in PD, BoP, and suppuration. Its combination with chemical disinfection, employing substances such as EDTA, tetracycline, hydrogen peroxide, and chlorhexidine, remains controversial [28,29]. Notably, no instances of implant loss or severe complications directly attributed to implantoplasty have been reported. Furthermore, Azzola et al. [30] showed that implantoplasty significantly reduced plaque coverage on the dental implant surface (16% plaque coverage compared to 65% of untreated dental implants). Histologically, a mild to moderate deposition of titanium particles in adjacent tissues, accompanied by localized inflammatory cell infiltrate, was observed 12 weeks post-operatively. Controlling the overheating of neighboring bone tissue during implantoplasty through standard cooling measures was found to be straightforward and did not raise concerns regarding osseous thermal damage [28,29]. However, it is noteworthy that a significant reduction in implant wall thickness may occur after implantoplasty; thus, narrow- or standard-diameter implants may experience variable weakening following the procedure. In particular, a single, narrow posterior implant undergoing implantoplasty is considered at a greater risk of mechanical complications compared to a three-unit bridge supported by two standard-diameter implants in the anterior region [28,29].

Surgical treatment of peri-implantitis comprises both resective and regenerative approaches [14].

Open flap debridement is recommended when infra-osseous defects are minimal and the quality of soft tissue is adequate. While effective in reducing PD, BoP, and suppuration by removing granulation tissue and decontaminating the exposed implant surface, this method exhibits poor predictability and clinical stability at the 5-year follow-up [25].

Both resective and regenerative surgical peri-implantitis treatments could significantly modify the peri-implantitis microbial profile due to the immediate change of peri-implantitis site anatomy [14].

Resective therapy is indicated for supracrestal bone defects with exposed threads in aesthetically non-demanding areas. This approach involves the reduction/removal of pathological peri-implant pockets, placement of apical mucosal flaps, bone recontouring, and implantoplasty [31]. Despite this, resective surgery does not exhibit a robust therapeutic effect for peri-implantitis, and the adjunctive use of local chemical decontamination, either with chloramines, tetracycline hydrochloride, and chlorhexidine gluconate, or with hydrogen peroxide, citric acid, and sodium chloride, has not demonstrated superior therapeutic effects [32].

Regenerative treatment for peri-implantitis shows greater improvements in radiographic bone fill and CAL compared to open flap debridement. However, it is less effective in reducing peri-implant soft tissue inflammation and does not significantly improve mucosal recession levels [32,33,34]. Nevertheless, the limitations and predictability of regenerative techniques, as well as the stability of outcomes, remain unclear, given that most studies have a follow-up of no more than 12 months [32,35,36,37,38].

Ultimately, implant removal is indicated when implant mobility is not related to occlusal trauma, bone loss exceeds two-thirds of the implant length, there is a failure to control repeated peri-implant abscesses associated with advanced bone loss, and when acceptable patient aesthetics cannot be achieved even with additional procedures [39].

Peri-implantitis treatment can reduce the total microbial load, and even if it does not eradicate predominant bacteria, it decreases the bacterial species that compete with the red complex for the peri-implant niches, probably further favoring high-virulence species capable of recolonizing peri-implantitis sites after treatment more quickly compared to the less virulent bacterial species [34].

Both surgical and non-surgical peri-implantitis mechanical debridement, even with different adjunctive chemical or physical therapies, do not significantly reduce tissue-invasive bacteria, particularly *Fusobacterium nucleatum*, which showed high levels even after peri-implantitis treatments and seemed not affected by clinical or other microbial profile variations [14]. These findings underscore the need for innovative peri-implantitis treatments capable of controlling persistent and tissue-invasive bacteria in the peri-implant environment.

## Data Availability

Data are available on PubMed/MEDLINE, Scopus, and Web of Science databases.
